# Clinical outcomes and mortality risk of in-hospital cardiac arrest in patients with acute myocardial infarction complicated by cardiogenic shock

**DOI:** 10.3389/fcvm.2025.1663933

**Published:** 2025-11-13

**Authors:** Jing Zhang, Chengcheng Shao, Jiajia Zhu, Jiang Li, Liying Chen

**Affiliations:** 1 Cardiac Critical Care Center Ward I, Beijing Anzhen Hospital, Capital Medical University, Beijing, China; 2 Cardiac Critical Care Center Ward Ⅱ, Beijing Anzhen Hospital, Capital Medical University, Beijing, China; 3 Department of Cardiology, Beijing Anzhen Hospital, Capital Medical University, Beijing, China

**Keywords:** cardiogenic shock, in-hospital cardiac arrest, reversible cardiac arrest, non-reversible cardiac arrest, acute myocardial infarction

## Abstract

**Background:**

This study investigated the clinical characteristics of in-hospital cardiac arrest (IHCA) in patients with acute myocardial infarction (AMI) complicated by cardiogenic shock and assessed the related in-hospital and post-discharge mortality.

**Methods:**

This study included 148 patients with AMI complicated by cardiogenic shock who were admitted to the Cardiac Critical Care Center, Department of Cardiology, Beijing Anzhen Hospital, Capital Medical University between September 1, 2021 and July 31, 2024. Study participants were divided into two groups according to the occurrence of IHCA (IHCA group, *n* = 62 and control group, *n* = 86). The primary endpoint was in-hospital mortality, whereas secondary endpoints included in-hospital complications (e.g., ischemic stroke, hemorrhagic stroke, gastrointestinal bleeding, and lower limb ischemia), 30-day post-discharge mortality, and 1-year post-discharge mortality. The association between IHCA and in-hospital mortality was assessed by Cox regression analysis, and post-discharge mortality risks were evaluated by modified Poisson regression analysis.

**Results:**

IHCA was associated with significantly higher in-hospital mortality (61.3% vs. 29.8%, *P* < 0.001), 30-day mortality (67.7% vs. 39.3%, *P* = 0.001), and 1-year mortality (71.0% vs. 40.5%, *P* = 0.001) compared with the control group. Cox regression analysis showed that IHCA increased the risk of in-hospital mortality [hazard ratio [HR] 2.064, 95% confidence interval [CI] 1.180–3.609, *P* = 0.011]. The relative risks of death within 30 days and 1 year post-discharge were 1.606 (95% CI 1.172–2.201, *P* = 0.003) and 1.644 (95% CI 1.216–2.222, *P* = 0.001), respectively. IHCA patients with non-reversible cardiac arrest had a higher 30-day mortality risk [relative risk (RR) 1.599, 95% CI 1.118–2.286, *P* = 0.010] than those with reversible cardiac arrest, although no significant difference was observed in the risk of 1-year mortality (RR 1.369, 95% CI 0.975–1.922, *P* = 0.070).

**Conclusions:**

IHCA increases in-hospital, 30-day, and 1-year mortality risks in patients with AMI complicated by cardiogenic shock. Non-reversible cardiac arrest notably increases the risk of death within 30 days post-discharge.

## Introduction

Acute myocardial infarction (AMI) can lead to severe complications such as cardiogenic shock (CS) and cardiac arrest (CA), which are both associated with high fatality rates. The mortality rate of AMI with CS ranges from 30% to 40% (1), while limited research has examined the co-occurrence of CS and CA (2). The 2022 Society for Cardiovascular Angiography and Interventions (SCAI) Shock stage classification underscores the increased mortality risk from CA across CS stages. Notably, the combination of CS and CA significantly increases mortality, with risks linked to mechanical circulatory support (MCS) and multiorgan failure (3). Brain damage and organ failure, rather than cardiac dysfunction, are the primary causes of death following CA.

Cardiac arrest is categorized by the setting in which it occurs, as out-of-hospital CA (OHCA) or in-hospital CA (IHCA) (4). Outcomes of CA are influenced by factors such as witnessed events, arrest rhythms (e.g., defibrillable rhythms), bystander cardiopulmonary resuscitation (CPR) availability, and CPR quality and duration (5). These factors vary between OHCA and IHCA, contributing to differences in clinical results (6). In China, OHCA affects approximately 1.05 million individuals annually, with a survival-to-discharge rate of only 1%. IHCA, although less frequent (1.75% incidence), has a 9.1% survival rate, which remains lower than global averages (7). This stark disparity highlights the need to further explore the characteristics and risk factors for IHCA-related mortality in AMI patients with CS.

## Methods

### Study population

**Figure 1:**
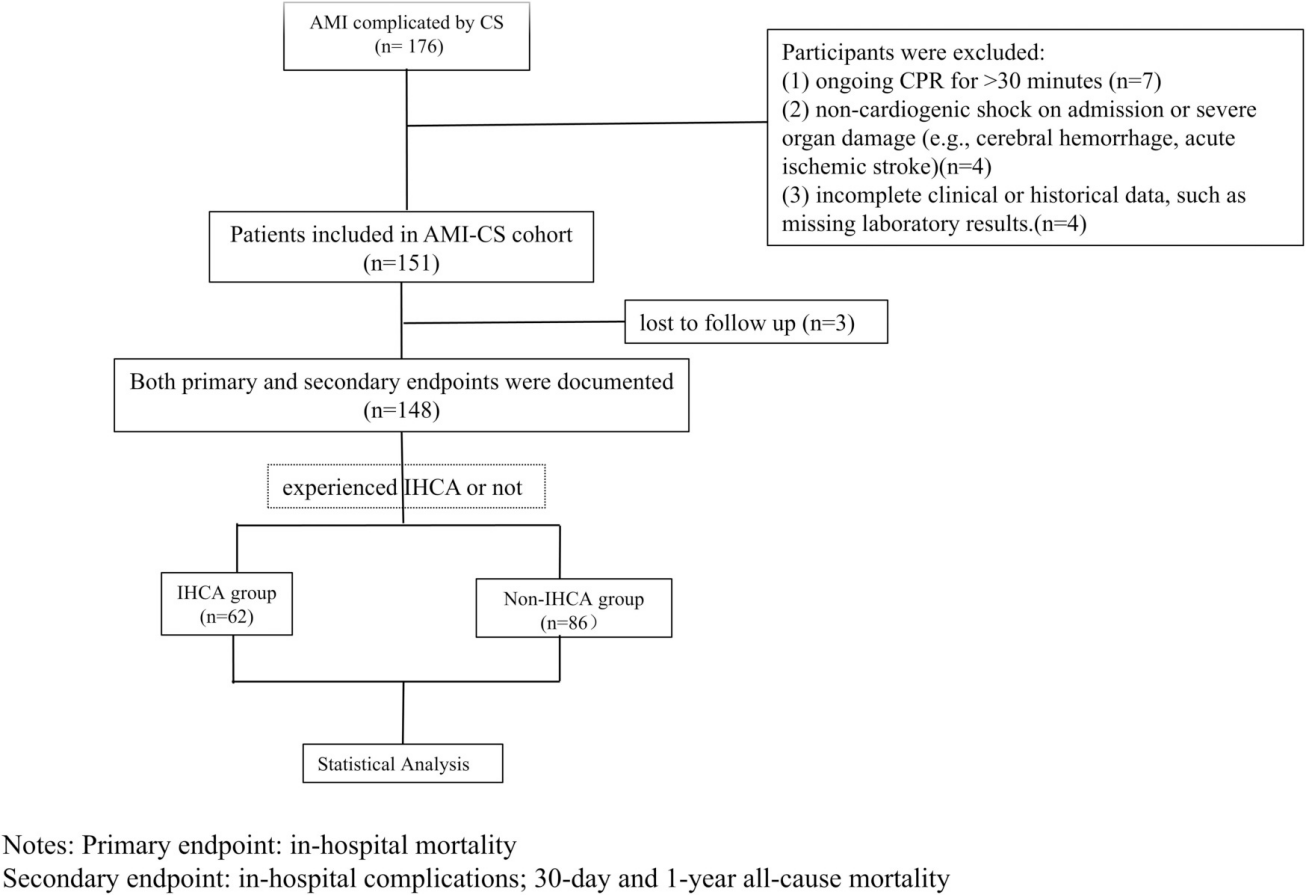
Workflow of patients selection.

This observational study enrolled patients diagnosed with AMI complicated by CS who were admitted to the Cardiac Critical Care Center, Beijing Anzhen Hospital, Capital Medical University between September 1, 2021 and July 31, 2024. The workflow of patient selection is illustrated in Figure 1. Participants were divided into two groups based on whether they experienced IHCA. The IHCA group was further subdivided by cardiac arrest rhythm: ventricular fibrillation or pulseless ventricular tachycardia, categorized as reversible cardiac arrest (ventricular fibrillation cardiac arrest, VFCA), and non-defibrillable cardiac arrest (non-ventricular fibrillation cardiac arrest, NVFCA), characterized by cardiac standstill or electromechanical dissociation. The clinical characteristics and outcomes of each subgroup were recorded. The study received ethical approval from our hospital's institutional review board and was performed in compliance with the Declaration of Helsinki. The requirement of informed consent was waived due to the observational design of the study.

### Inclusion and exclusion criteria

Patients were included if they met the following criteria: (1) diagnosis of AMI with CS and (2) age ≥18 years. The exclusion criteria were: (1) ongoing CPR for >30 min, (2) non-cardiogenic shock on admission or severe organ damage (e.g., cerebral hemorrhage, acute ischemic stroke), and (3) incomplete clinical or historical data, such as missing laboratory results.

### Diagnostic criteria

#### AMI

Clinical symptoms: Typical chest pain, pressure, or discomfort in the retrosternal or precordial region, potentially radiating to the neck, jaw, shoulders, or back. Electrocardiography (ECG): Dynamic ST-T changes in at least two contiguous leads, including ST-segment elevation or depression. Laboratory tests: Elevated myocardial injury markers (e.g., high-sensitivity troponin T/I and creatine kinase isoenzyme) with dynamic changes.

#### CS

Hypotension: Systolic blood pressure (SBP) ≤90 mmHg for >30 min or requirement of vasoactive agents to maintain SBP >90 mmHg (after ruling out hypovolemia).

Peripheral hypoperfusion: Cool, clammy skin, oliguria, altered mental status, lactate >2 mmol/L, or reliance on inotropes/circulatory support devices.

#### IHCA

IHCA was defined by an event clearly documented in the medical records as ventricular tachycardia (VT), ventricular fibrillation (VF), or cardiac arrest occurring while a patient was in the Cardiac Critical Care Center for monitoring and treatment, excluding those episodes that occurred during revascularization procedures.

### Treatment strategies

Patient enrollment and treatment were centrally managed by a Shock Team, consisting of interventional cardiologists, cardiac intensive care unit (CICU) physicians, and cardiac surgeons.

The interventional cardiologists were responsible for assessing indications for emergency coronary angiography and percutaneous intervention, as well as performing these procedures. Cardiac surgeons evaluated indications for coronary artery bypass grafting (CABG), heart transplantation, and left ventricular assist device (LVAD) implantation, and carried out the corresponding surgical operations.

CICU physicians assessed the patients’ clinical parameters and hemodynamic status, and made decisions regarding inotropic and vasoactive drug support. They also evaluated patients' oxygenation and metabolic circulation status to determine the need for mechanical ventilatory support and continuous renal replacement therapy (CRRT). Furthermore, they managed the escalation or de-escalation of temporary mechanical circulatory support (tMCS) and the weaning from tMCS. Escalation to MCS was considered when pharmacological therapy alone failed to improve perfusion or adequately reduce cardiac load. Decisions regarding tMCS weaning were based on recovery of end-organ function and the status of tMCS-associated complications.

### Data collection

Demographic and clinical data were collected, including age, gender, medical history, and discharge outcomes. Initial assessments within 15 min of admission documented heart rate, blood pressure, and laboratory results such as blood gas analysis and renal function tests. Information on the use of MCS, including intra-aortic balloon pump (IABP) and extracorporeal membrane oxygenation (ECMO), was gathered, alongside MCS duration. Vasoactive inotropic score (VIS) and oxygen therapy modalities (e.g., room air, nasal cannula, mask, non-invasive ventilation, or intubation) were also recorded. Initial echocardiographic findings, such as left ventricular ejection fraction (LVEF), were documented. The use of revascularization methods, including percutaneous coronary intervention (PCI), percutaneous coronary balloon angioplasty (PTCA), or coronary artery bypass grafting (CABG), also was recorded. Additionally, in-hospital complications, including ischemic stroke, hemorrhagic stroke, gastrointestinal bleeding, lower limb ischemia, pulmonary infection, and renal failure requiring continuous renal replacement therapy, were tracked.

### Endpoints

The primary endpoint was in-hospital mortality. Secondary endpoints included in-hospital complications (e.g., acute ischemic stroke, hemorrhagic stroke, gastrointestinal bleeding, lower limb ischemia, etc.), as well as all-cause mortality measured at 30 days and 1 year post-discharge.

### Statistical analysis

The Kolmogorov–Smirnov test was used to assess normality of data distributions. The Kruskal–Wallis test was used for comparisons of non-normally distributed variables, while the *t*-test was applied for comparisons of normally distributed data. Categorical variables were analyzed using the chi-square test or Fisher's exact test. Cox regression analysis was conducted to determine the association between the risk of in-hospital mortality and IHCA, and the survival curves were generated. Modified Poisson regression was performed to evaluate mortality risk at 30 days and 1 year post-discharge. Variables selected based on univariate analysis and prior studies were included in the two regression models to control for confounders.

Two different models were used in the study. Model 1 adjusted for age and gender. Model 2 adjusted for age, gender, weight, length of hospital stay, smoking status, VIS, hypertension, diabetes, and chronic kidney disease. Hazard ratios (HRs), relative risks (RRs), and 95% confidence intervals (CIs) were calculated. Statistical analyses were performed using SPSS (IBM SPSS Inc., Chicago, IL, version 26.0) and R software (version 4.1.2), with a two-sided *P*-value <0.05 reflecting statistical significance.

## Results

### Baseline characteristics

The study included 148 patients, of whom 62 experienced IHCA, resulting in an IHCA incidence rate of 41.9%. A comparative analysis of baseline characteristics between the IHCA and non-IHCA groups is provided in Table 1. The percentage of male patients was significantly larger in the IHCA group than in the non-IHCA group (80.6% vs. 62.6%, *P* < 0.05). No statistically significant differences were observed between the groups regarding medical history (e.g., hypertension, diabetes, chronic kidney disease, stroke, prior coronary interventions, or coronary artery bypass grafting; *P* > 0.05). Similarly, other parameters such as ST-segment elevation myocardial infarction (STEMI), pre-hospital thrombolysis, heart rate, blood pressure on admission, and body weight did not differ significantly between the groups (*P* > 0.05). However, LVEF was markedly higher in the non-IHCA group than in the IHCA group [40.00% [33.00%, 50.00%] vs. 30.00% [22.00%, 38.75%], *P* < 0.001]. Additionally, the lactate level [3.45 mmol/L [1.83, 8.95 mmol/L] vs. 2.70 mmol/L [1.70, 4.50 mmol/L], *P* < 0.05] and VIS [10.00 [0.00, 28.00] vs. 0.95 [0.00, 20.00], *P* < 0.05] were significantly higher in the IHCA group than in the non-IHCA group.

**Table 1:** Comparison of baseline characteristics between the IHCA and non-IHCA groups.

Baseline characteristic	All (*N* = 148)	Non-IHCA group (*n* = 86)	IHCA group (*n* = 62)	*P* value
Age (years)	61.08 ± 11.87	61.38 ± 12.16	60.66 ± 11.55	0.716
Male (%)	104 (70.3)	54 (62.8)	50 (80.6)	0.031
Smoking (%)	76 (51.4)	40 (46.5)	36 (58.1)	0.222
History (%)				
Hypertension (%)	87 (58.8)	52 (60.5)	35 (56.5)	0.749
Diabetes (%)	65 (43.9)	36 (41.9)	29 (46.8)	0.670
Chronic kidney disease (%)	8 (5.4)	6 (7.0)	2 (3.2)	0.531
Ischemic stroke history (%)	22 (14.9)	9 (10.5)	13 (21.0)	0.124
Hemorrhagic stroke history (%)	0	0	0	NA
MI history (%)	15 (10.1)	6 (7.0)	9 (14.5)	0.221
PCI (%)	19 (12.8)	7 (8.1)	12 (19.4)	0.078
CABG (%)	3 (2.0)	1 (1.2)	2 (3.2)	0.774
MI (%)				
Non-ST elevated MI (%)	30 (20.3)	18 (20.9)	12 (19.4)	0.811
ST elevated MI (%)	118 (79.7)	68 (79.1)	50 (80.6)	
Pre-hospital thrombolysis (%)	15 (10.1)	6 (7.0)	9 (14.5)	0.221
Clinical manifestation				
Heart rate (beats per minute)	97.84 ± 25.58	98.48 ± 22.75	96.97 ± 29.23	0.725
Systolic pressure (mmHg)	96.86 ± 23.72	98.72 ± 24.79	94.29 ± 22.07	0.264
Diastolic pressure (mmHg)	63.02 ± 15.50	64.12 ± 14.78	61.50 ± 16.45	0.313
Weight (kg)	71.64 ± 11.51	70.85 ± 10.01	72.73 ± 13.33	0.327
Lactic acid (mmol/L)	3.20 [1.70, 5.65]	2.70 [1.70, 4.50]	3.45 [1.83, 8.95]	0.018
EF	35.00 (28.00, 46.50)	40.00 (33.00, 50.00)	30.00 (22.00, 38.75)	<0.001
Vasoactive inotropic score	4.70 (0.00, 24.75)	0.95 (0.00, 20.00)	10.00 (0.00, 28.00)	0.026

EF, ejection fraction; PTCA, percutaneous coronary balloon angioplasty; PCI, percutaneous coronary intervention; CABG, coronary artery bypass grafting.

### Treatment strategies and outcomes

The treatment interventions and clinical outcomes among the study population are summarized in Table 2. While the overall use of MCS did not differ significantly between the groups, a distinction was apparent in the types of support utilized. Combined IABP and ECMO therapy was more frequently employed in the IHCA group (45.8%), whereas IABP alone was predominantly used in the non-IHCA group (66.2%). The ECMO duration was also longer in the IHCA group than in the non-IHCA group. Moreover, a greater proportion of patients in the IHCA group required ventilator support, both non-invasive and invasive, and their mechanical ventilation durations were extended compared with those in the non-IHCA group. Among the observed in-hospital complications, gastrointestinal hemorrhage occurred significantly more often in the IHCA group than in the non-IHCA group, whereas the rates of ischemic stroke, hemorrhagic stroke, lower limb ischemia, and acute renal injury requiring continuous renal replacement therapy (CRRT) were comparable between the groups (all *P* > 0.05). Mortality outcomes revealed a significantly higher in-hospital death rate for the IHCA group compared with the non-IHCA group (61.3% vs. 29.8%, *P* < 0.001). Similar trends were observed at 30 days (67.7% vs. 39.3%, *P* = 0.001) and at 1 year post-discharge (71.0% vs. 40.5%, *P* = 0.001).

**Table 2:** Comparison of in-hospital complications and mortality between the IHCA and non-IHCA groups.

Treatments and in-hospital complications	All (*N* = 148)	Non-IHCA group (*n* = 86)	IHCA group (*n* = 62)	*P* value
Treatment				
Mechanical circulatory assist (%)	117 (79.1)	70 (81.4)	47 (75.8)	0.359
Mechanical circulatory assist category				
IABP assist (%)	65 (55.6)	47 (67.1)	18 (38.3)	0.002
ECMO assist (%)	20 (17.1)	13 (18.6)	7 (14.9)	
IABP combined with ECMO assist (%)	32 (27.3)	10 (14.3)	22 (46.8)	
Ventilation assist (%)	95 (65.1)	47 (54.7)	48 (77.4)	0.012
IABP duration (days)	2.00 (0.00, 7.00)	2.50 (0.00, 7.00)	2.00 (0.00, 5.75)	0.290
ECMO duration (days)	0.00 (0.00, 2.00)	0.00 (0.00, 1.00)	0.00 (0.00, 3.00)	0.009
In-hospital complications				
Acute renal failure requiring CRRT (%)	26 (17.6)	12 (14.0)	14 (22.6)	0.297
Gastrointestinal bleeding (%)	28 (18.9)	8 (9.3)	20 (32.3)	0.001
Hemorrhagic stroke (%)	4 (2.7)	1 (1.2)	3 (4.8)	0.4
Ischemic stroke (%)	14 (9.5)	7 (8.1)	7 (11.3)	0.752
Lower limb ischemia (%)	8 (5.4)	6 (7.0)	2 (3.2)	0.509
Blood stream infection (%)	4 (2.7)	2 (2.3)	2 (3.2)	1.000
Pulmonary infection (%)	59 (40.4)	31 (36.9)	28 (45.2)	0.404
In-hospital mortality (%)	63 (43.2)	25 (29.8)	38 (61.3)	<0.001
30-day mortality (%)	75 (51.4)	33 (39.3)	42 (67.7)	0.001
1-year mortality (%)	78 (53.4)	34 (40.5)	44 (71.0)	0.001

IABP, intra-aortic balloon pulsation; ECMO, extracorporeal membrane oxygenation; CRRT, continuous renal replacement therapy.

### Coronary angiographic findings

The detailed results of coronary angiography are presented in Table 3. Isolated left main coronary lesions were more common in the IHCA group than in the non-IHCA group (38.0% vs. 17.1%, *P* = 0.015), as were left main coronary lesions combined with three-vessel disease (17.7% vs. 4.7%, *P* = 0.020). No significant differences between groups were observed regarding involvement of the left anterior descending, circumflex, or right coronary arteries (*P* > 0.05). The IHCA group had a significantly lower revascularization rate than the non-IHCA group (62.9% vs. 85.7%, *P* < 0.05).

**Table 3:** Comparison of coronary lesions between the IHCA and non-IHCA groups.

Coronary features and convention	All group	Non-IHCA group	IHCA group	*P* value
Single left main lesion (%)	32 (25.4)	13 (17.1)	19 (38.0)	0.015
Left main combined with three lesions (%)	15 (10.1)	4 (4.7)	11 (17.7)	0.020
Anterior descendant (%)	101 (80.2)	62 (81.6)	39 (78.0)	0.791
Circumflex branch (%)	70 (47.3)	41 (47.7)	29 (46.8)	1.000
Right coronary artery (%)	64 (50.8)	35 (46.1)	29 (58.0)	0.258
Revascularization (%)	111 (76.0)	72 (85.7)	39 (62.9)	0.003
Revascularization method (%)				
PTCA or PCI (%)	80 (72.1)	44 (62.0)	36 (90.0)	0.004
CABG (%)	27 (24.3)	24 (33.8)	3 (7.5)	
PTCA/PCI combined with CABG (%)	3 (2.7)	3 (4.2)	0 (0.0)	

PTCA, percutaneous coronary balloon angioplasty; PCI, percutaneous coronary intervention; CABG, coronary artery bypass grafting.

### IHCA subgroup analysis: VFCA vs. NVFCA

Among the 62 IHCA patients, 47 had VFCA, and 15 had NVFCA. No significant differences were identified between these subgroups in terms of in-hospital complications such as acute renal injury requiring CRRT, gastrointestinal hemorrhage, ischemic stroke, hemorrhagic stroke, lower limb ischemia, bloodstream infection, or pulmonary infection (*P* > 0.05). Similarly, the use and duration of MCS (IABP and ECMO) were comparable between the VFCA and NVFCA subgroups (*P* > 0.05). In-hospital mortality rates also did not differ significantly between the VFCA and NVFCA subgroups (80.0% vs. 55.3%, *P* > 0.05). However, the 30-day post-discharge mortality rate was notably higher in the NVFCA group than in the VFCA group (93.3% vs. 59.6%, *P* < 0.05), whereas no significant difference was observed in the 1-year mortality rate between the subgroups (93.3% vs. 63.8%, *P* > 0.05; Table 4).

**Table 4:** Comparison between VFCA and NVFCA subgroups.

Outcomes	IHCA group (*n* = 62)	NVFCA (*n* = 15)	VFCA (*n* = 47)	*P* value
In-hospital complications				
Acute renal failure requiring CRRT (%)	14 (22.6)	6 (40.0)	8 (17.0)	0.134
Gastrointestinal bleeding (%)	20 (32.3)	6 (40.0)	14 (29.8)	0.675
Hemorrhagic stroke (%)	3 (4.8)	2 (13.3)	1 (2.1)	0.285
Ischemic stroke (%)	7 (11.3)	1 (6.7)	6 (12.8)	0.856
Lower limb ischemia (%)	2 (3.2)	1 (6.7)	1 (2.1)	0.978
Blood stream infection (%)	2 (3.2)	0 (0.0)	2 (4.3)	1.000
Pulmonary infection (%)	28 (45.2)	8 (53.3)	20 (42.6)	0.665
Mechanical circulatory assist (%)	56 (90.3)	14 (93.3)	42 (89.4)	1.000
Mechanical circulatory assist				
IABP duration (days)	2.00 (0.00, 5.75)	2.00 (0.00, 5.00)	2.00 (0.00, 5.50)	0.666
ECMO duration (days)	0.00 (0.00, 3.00)	0.00 (0.00, 1.00)	1.00 (0.00, 3.00)	0.144
In-hospital mortality (%)	38 (61.3)	12 (80.0)	26 (55.3)	0.160
30-day mortality (%)	42 (67.7)	14 (93.3)	28 (59.6)	0.034
1-year mortality (%)	44 (71.0)	14 (93.3)	30 (63.8)	0.062

IHCA, in-hospital cardiac arrest; VFCA, cardiac arrest manifested as pulseless ventricular tachycardia and ventricular fibrillation that is cardiovertable; NVFCA, cardiac arrest that cannot be defibrillated as manifested by cardiac stationary or electromechanical separation; IABP, intra-aortic balloon pulsation; ECMO, extracorporeal membrane oxygenation; CRRT, continuous renal replacement therapy.

### Mortality risk analysis

**Figure 2:**
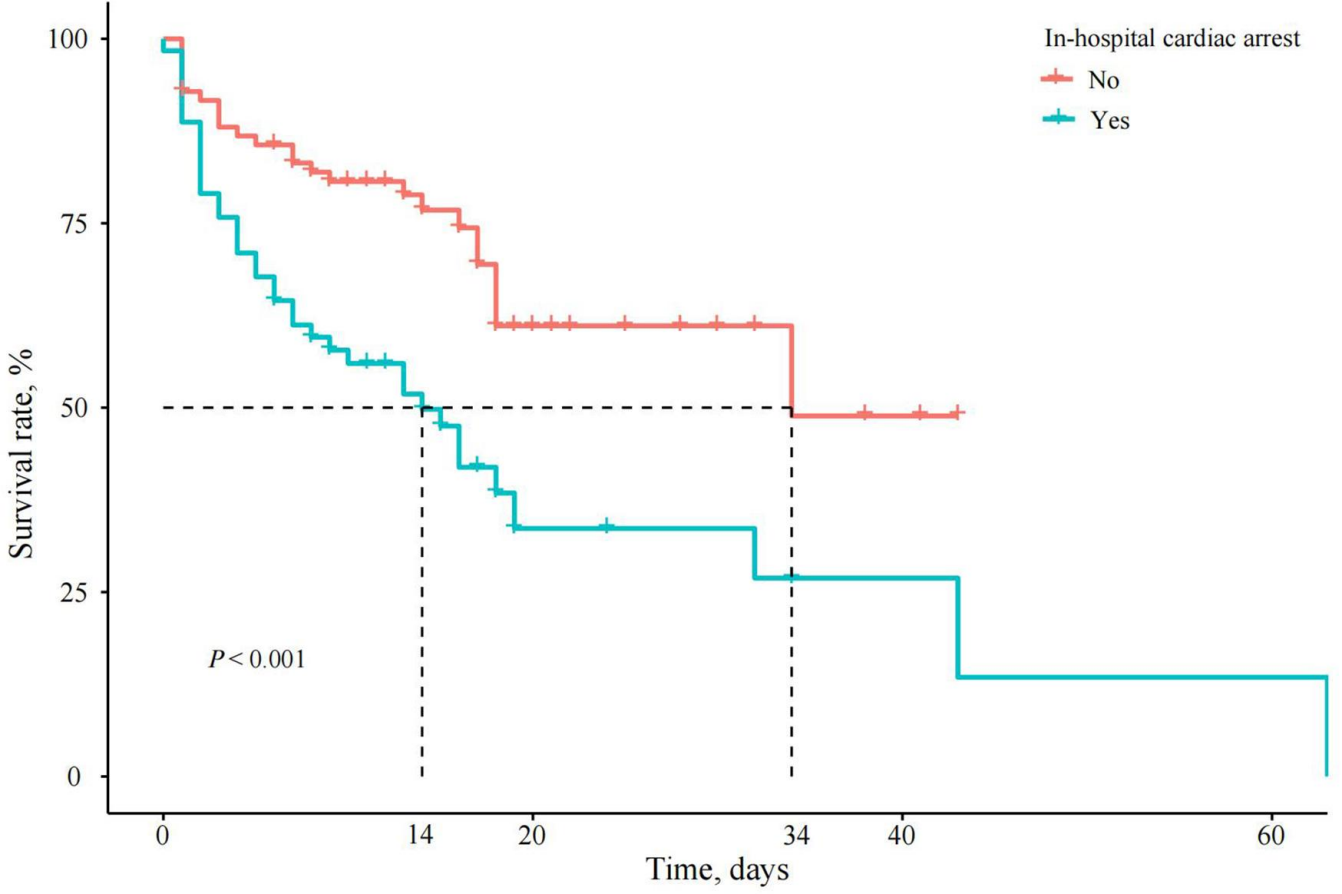
Survival curves of patients with AMI and CS with or without IHCA. In the IHCA group (red line), the survival rate plummeted to nearly 0% within 40 days, whereas the non-IHCA group (blue line) maintained consistently high survival rates throughout.

**Figure 3:**
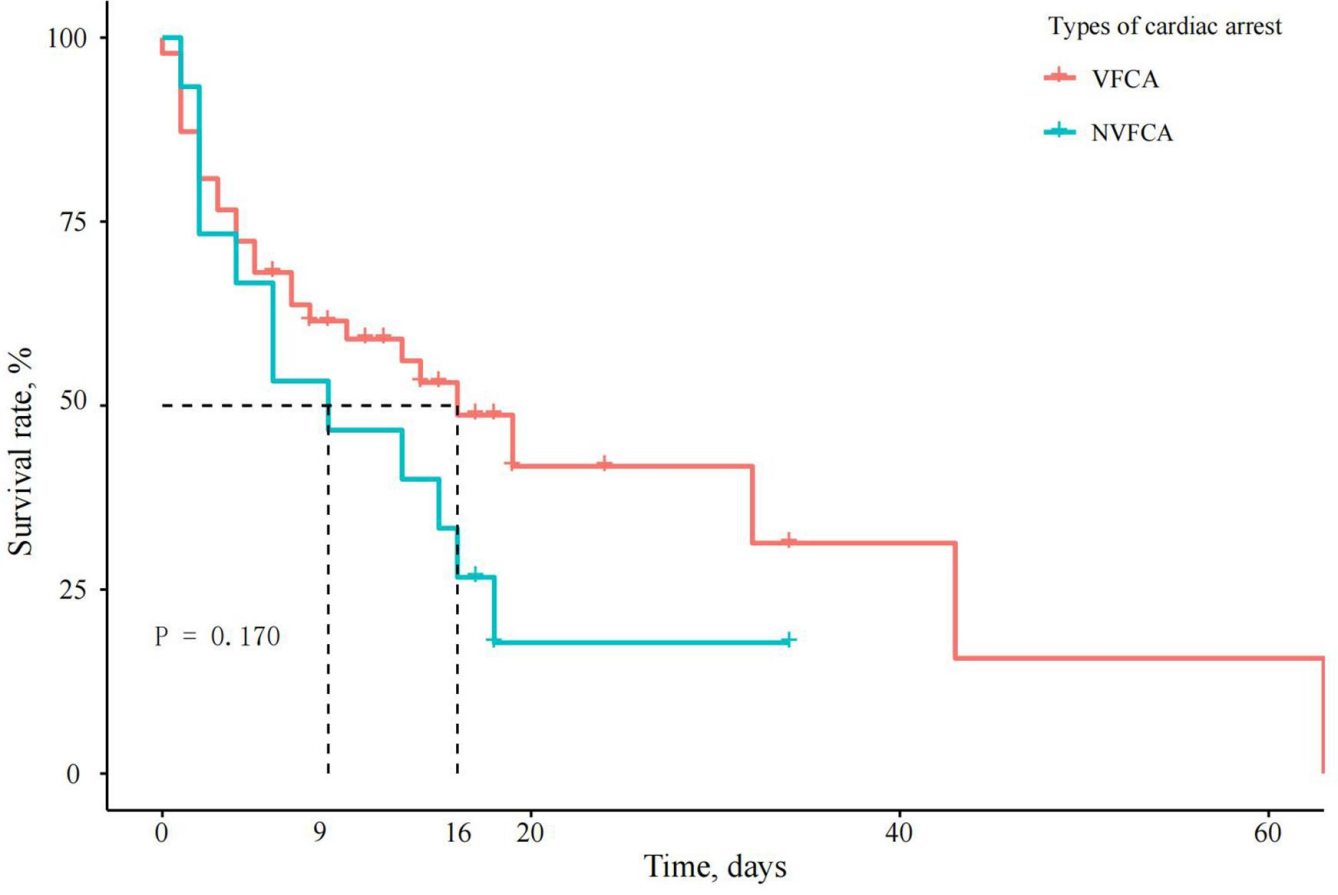
Survival curves of patients with AMI complicated by CS and different types of IHCA. The survival curves for VFCA and NVFCA had near-overlapping trajectories (*P* = 0.170), suggesting that the initial arrest rhythm type may not be an independent predictor of in-hospital mortality.

Cox regression analysis generated an adjusted HR of 2.064 (95% CI, 1.180–3.609, *P* = 0.011) for in-hospital mortality in the IHCA group. When the VFCA and NVFCA subgroups were compared, no significant difference in the in-hospital mortality risk was observed (HR 1.164, 95% CI 0.522–2.595, *P* > 0.05; Table 5). Survival curves illustrating these findings are presented in Figures 2, 3. Modified Poisson regression analysis revealed a significantly higher 30-day post-discharge mortality RR in the IHCA group than in the non-IHCA group (RR 1.606, 95% CI, 1.172–2.201, *P* = 0.003). When comparing the NVFCA to VFCA subgroups, the RR of death at 30 days was 1.599 in the NVFCA group (95% CI 1.118–2.286, *P* = 0.010). At 1 year, the IHCA group maintained a higher mortality risk (RR 1.644, 95% CI 1.216–2.222, *P* = 0.001), but no significant differences were found between the VFCA and NVFCA subgroups (RR 1.369, 95% CI 0.975–1.922, *P* = 0.070) (Table 6). These data suggests that initial arrest rhythm type may not be an independent predictor of in-hospital mortality.

**Table 5:** Associations of IHCA and its types with in-hospital mortality in patients with myocardial infarction.

Outcomes	IHCA and types	Model 1		Model 2	
		HR (95% CI)	*P* value		HR (95% CI)
In-hospital mortality	In-hospital cardiac arrest				
	No	Reference		Reference	
	Yes	2.805 (1.636–4.809)	<0.001	2.064 (1.180–3.609)	0.011
	Type				
	VFCA	Reference		Reference	
	NVFCA	1.501 (0.710–3.173)	0.288	1.164 (0.522–2.595)	0.711

**Table 6:** Associations of IHCA and its types with out-hospital mortality in patients with myocardial infarction.

Outcomes	IHCA and types	Model 1		Model 2	
		RR (95% CI)	*P* value		RR (95% CI)
30-day mortality	In-hospital cardiac arrest				
	No	Reference		Reference	
	Yes	1.729 (1.255–2.381)	0.001	1.606 (1.172–2.201)	0.003
	Type				
	VFCA	Reference		Reference	
	NVFCA	1.545 (1.165–2.050)	0.003	1.599 (1.118–2.286)	
1-year mortality	In-hospital cardiac arrest				0.001
	No	Reference		Reference	
	Yes	1.791 (1.318–2.434)	<0.001	1.644 (1.216–2.222)	
	Type				0.070
	VFCA	Reference		Reference	
	NVFCA	1.422 (1.090–1.855)	0.010	1.369 (0.975–1.922)	

Model 1 adjusted for age and sex. Model 2 further adjusted for hospitalization duration, smoking status, bodyweight, vascular remodeling, mean arterial pressure, VIS, and history of hypertension, diabetes, and chronic kidney disease.

VFCA, cardiac arrest manifesting as pulseless ventricular tachycardia and ventricular fibrillation that is cardiovertable; NVFCA, cardiac arrest that cannot be defibrillated as manifested by cardiac statioiknary or electromechanical separation.

## Discussion

CS is a clinical condition resulting from cardiac pump failure that leads to severe acute peripheral organ dysfunction and high mortality rates (8). The most frequent cause of CS following AMI is severe left-sided heart failure, typically after anterior myocardial infarction (9). Prior studies have explored the clinical and coronary features of patients with AMI and OHCA, identifying factors such as younger age, extensive infarction, left main coronary artery lesions, chronic total occlusion, and absence of calcium channel blocker use as significant contributors to OHCA (10). However, studies focusing on the clinical features of IHCA in AMI and CS patients remain limited. This study aimed to further investigate the prognosis and clinical features of such patients.

Our findings reveal that the incidence of IHCA in patients with AMI and CS was significantly higher than in those with ST-elevation myocardial infarction (STEMI) alone (11). Furthermore, patients in the IHCA group had a significantly lower LVEF than those in the non-IHCA group. Reduced LVEF is a well-established prognostic factor in AMI (12). Prior studies have shown that patients with LVEF ≤30% after AMI are at the highest risk for cardiac arrest within 1 month, with each 5% decrease in LVEF increasing the risk of cardiac arrest by 21% (13). Our findings corroborate this, emphasizing the value of LVEF as a critical predictor of mortality in this cohort.

The blood lactate level, which reflects the extent of anaerobic metabolism, is positively correlated with the severity of circulatory disturbances and mortality risk in shock (14). In our study, lactate levels were significantly higher in the IHCA group than in the non-IHCA group, indicating more severe circulatory failure in these patients. The elevated VIS in the IHCA group further suggests that these patients experience greater hemodynamic instability. VIS is an independent risk factor for in-hospital mortality in AMI and CS (15).

Treatment analysis showed that the proportion of patients who received non-invasive and invasive ventilation support was higher and the duration of mechanical ventilation was longer in the IHCA group than in the non-IHCA group. MCS use also was more frequent in the IHCA group, including greater reliance on a combination of IABP and ECMO, and longer ECMO durations than in the non-IHCA group. These results suggest that the more severe hemodynamic instability in the IHCA group require more intensive and prolonged treatment.

Revascularization plays a crucial role in the treatment of AMI with CS, as early revascularization has been shown to significantly reduce mortality (14). In our study, the IHCA group included a higher proportion of patients with complex coronary pathology, including isolated left main lesions and three-vessel disease, along with a lower revascularization rate than the non-IHCA group. This aligns with the findings of previous studies, which reported lower rates of coronary intervention in IHCA patients (16, 17). Further in-depth investigation is warranted to comprehensively elucidate the underlying reasons why revascularization was not performed in these cases.

IHCA was significantly associated with increased in-hospital mortality and higher mortality rates at 30 days and 1 year post-discharge in patients with AMI and CS. This finding is consistent with the results of other studies, demonstrating that IHCA in STEMI patients is a major determinant of poor outcomes (18). The higher mortality rate observed in the IHCA group is likely due to several factors, including lower LVEF, extensive coronary disease, lower revascularization rate, and more severe circulatory failure compared with the non-IHCA group.

In addition, we examined the differences between two types of IHCA: VFCA and NVFCA. Previous studies have shown that NVFCA is associated with worse survival outcomes compared with VFCA. Thompson et al. (19) observed a significantly higher survival rate among VFCA patients than among NVFCA patients. Similarly, a retrospective analysis by the Mayo Clinic (20) found that NVFCA was more common in patients with IHCA and was associated with a higher risk of in-hospital death. In our study, a higher proportion of patients in the IHCA group had VFCA, which is consistent with previous findings in AMI patients. However, the increased mortality risk associated with NVFCA was evident only in the 30-day post-discharge period, with no significant difference in 1-year mortality between the VFCA and NVFCA subgroups.

The relatively higher mortality in the NVFCA subgroup within 30 days could be attributed to the absence of other underlying conditions. Our study focused exclusively on patients with AMI, which itself carries a high mortality risk. The prognosis of patients with other underlying cardiac conditions, such as arrhythmogenic cardiomyopathy, idiopathic arrhythmia, or valvular disease, may differ, but these conditions were not considered in this analysis.

In summary, while it is widely accepted in clinical practice that IHCA is associated with a poor prognosis, there remains a lack of published clinical studies specifically addressing IHCA in patients with AMI complicated by CS—particularly in the current era of widespread availability of MCS. Poor clinical outcomes in these patients include higher in-hospital, 30-day, and 1-year mortality rates. Factors such as low LVEF, extensive coronary disease, and severe circulatory failure contribute to the increased mortality risk. Based on these clinical characteristics of the IHCA cohort, our findings suggest the need for enhanced management strategies, including timely revascularization and optimized MCS utilization, in high-risk patients exhibiting these features. Further studies are necessary to explore the underlying mechanisms and identify optimal strategies for managing IHCA in this population.

## Conclusion

Patients who experience IHCA following AMI and CS are at significantly higher risk for in-hospital mortality, as well as increased mortality at 30 days and 1 year post-discharge. These patients generally present with a lower LVEF and an elevated lactate level, often exhibiting more severe coronary artery disease, such as left main lesions and multivessel disease. When examining IHCA subtypes, NVFCA was linked to higher 30-day mortality compared to VFCA. These findings underscore the critical need for early intervention and tailored management strategies in this high-risk patient group.

### Limitations

This study has several limitations. First, it was an observational study, and no experimental interventions were applied. Randomized controlled trials could provide more comprehensive insights into IHCA occurrence and progression. Second, the study was conducted at a single-center, which may introduce biases related to institutional differences in treatment strategies. Variations in patient selection and management across multiple centers could improve the generalizability of the results. Thirdly, microaxial flow pump use has not yet been widely adopted in clinical practice in China. Currently, the tMCS options available in our clinical practice include IABP and ECMO, and thus, this observational study was conducted within this context. Finally, the relatively small sample size and the limited follow-up data may reduce the generalizability of the findings. A larger sample with more comprehensive follow-up would strengthen the robustness of these results.

## Data Availability

The raw data supporting the conclusions of this article will be made available by the authors, without undue reservation.
